# How well do large language models answer postoperative lumbar fusion questions? A blinded comparative analysis of accuracy, quality, readability, and safety-related content

**DOI:** 10.3389/fsurg.2026.1932133

**Published:** 2026-10-01

**Authors:** Ahmet Kürşat Kara, Ozan Işık

**Affiliations:** 1 Department of Neurosurgery, Antalya City Hospital, Antalya, Türkiye; 2 Department of Neurosurgery, TC Saglik Bakanligi Denizli Servergazi Devlet Hastanesi, Denizli, Türkiye

**Keywords:** large language models, lumbar fusion, patient safety, postoperative patient education, readability

## Abstract

**Background:**

Patients recovering from lumbar fusion increasingly seek guidance from large language model (LLM) chatbots when their surgeon is unavailable, but the accuracy, completeness, and safety-related content of such responses have not been compared across models for the postoperative period.

**Objective:**

To compare the accuracy, quality, safety-related content, and readability of responses from ChatGPT, Gemini, and Claude to frequently asked postoperative lumbar fusion questions, and to identify the question types for which responses are weakest.

**Methods:**

Thirty questions, compiled by three neurosurgeons and grouped into six clinical categories, were submitted to each model, each with a standardized, patient-oriented prompt requesting warning signs. Ninety anonymized, randomized responses were rated independently by three blinded neurosurgeons for accuracy and four quality subdomains (clarity, completeness, relevance, and patient safety) on 4-point scales. Readability (Flesch–Kincaid, Flesch Reading Ease, and Gunning Fog), word count, and responses meeting an operational screening criterion for potential harm were also assessed. Models were compared with Friedman and Holm-corrected pairwise tests.

**Results:**

Accuracy was high for all models without significant differences. Quality, clarity, completeness, relevance, and patient safety differed globally, but effect sizes were small (Kendall's *W*: 0.10–0.21) and, after correction across the six outcome families, only quality and completeness remained significant, with Gemini scoring lower than ChatGPT and Claude. Gemini had the most favorable readability. ChatGPT responses were the shortest (mean 294 vs. 462 and 445 words); length was unrelated to completeness or readability. Two Gemini responses (6.7%) and none from ChatGPT or Claude met a *post hoc* operational screening criterion for potential harm (a score of ≤2 on accuracy or patient safety from at least one of three raters, without concurrence from the other two); no response was adjudicated as actually harmful. In exploratory question- and category-level analyses, responses tended to be rated highest for standardized questions and lowest for those requiring individualized judgment or interpretation of wound-related symptoms.

**Conclusion:**

Responses from contemporary LLMs to common postoperative lumbar fusion questions were generally rated as accurate, with small between-model differences and a model-level divergence between readability and completeness. Because safety-related content was explicitly prompted and no patient outcomes were assessed, these findings describe the quality of safety-related information rather than clinical safety. LLMs may support postoperative patient education but should not replace physician guidance, particularly for warning signs and patient-specific recovery decisions.

## Introduction

Lumbar fusion is among the most commonly performed procedures in neurosurgical practice, especially for patients with degenerative spinal disease, segmental instability, or recurrent disc pathology (1, 2). Surgeons today have access to better instrumentation and more refined perioperative protocols than ever before yet managing a patient in the weeks following lumbar fusion is rarely simple (3). This is where patient information becomes essential. When patients receive guidance that is clear, accurate, and easy to act on, adherence improves and so does their satisfaction with the overall recovery experience (4, 5).

In the postoperative period following lumbar fusion surgery, patients frequently seek information on pain management, mobilization, return to daily activities, recognition of potential complications, and expectations regarding surgical outcomes (6). However, the diversity of available information, along with occasional inconsistencies, may create confusion for patients. Inadequate or misinterpreted information can lead to poor treatment adherence, unnecessary anxiety, and, in some cases, delayed recognition of serious complications (7, 8). With the growing influence of the digital era, patients are increasingly turning to online platforms to access health-related information (9). While this trend has the potential to enhance patient engagement and health literacy, the accuracy, timeliness, and appropriateness of online content for patient-level understanding remain highly variable (10, 11). Specifically for complex interventions such as lumbar fusion surgery, the reliability of postoperative information is of paramount importance for patient safety (12).

During the preoperative decision-making process, patients typically reach their final decision through direct, face-to-face consultation with their physician, placing the highest level of trust in healthcare professionals at this stage (13). However, in the postoperative period, patients may not always have immediate access to their physicians. This situation, specifically in relation to daily activities, pain management, and potential signs of complications, increases patients’ need for prompt guidance and directs them to easily accessible digital resources, including artificial intelligence-based systems (14). Therefore, the accuracy and reliability of information provided by artificial intelligence become even more critical, especially in the postoperative period, with direct implications for patient safety (15).

In recent years, artificial intelligence-based large language models (LLMs) have demonstrated significant advancement in the fields of health communication and patient education (16, 17). Systems such as ChatGPT, Gemini, and Claude can generate rapid, context-aware, and human-like responses to user queries (18). These models have the potential to bridge the gap between patients and medical information by presenting complex clinical content in a more accessible and structured manner (19).

However, the performance of these systems in areas requiring a high degree of clinical precision—such as lumbar fusion surgery—has not yet been sufficiently elucidated (20, 21). In the current literature, studies that comprehensively evaluate the accuracy, completeness, patient safety, and readability of responses generated by artificial intelligence-based systems to postoperative patient queries remain limited (22, 23). Moreover, although head-to-head comparisons of ChatGPT, Gemini, and Claude exist for medical examination questions (24), and Lang et al. compared two models on lumbar fusion questions with expert raters (20), no study has compared three current-generation models on postoperative lumbar fusion questions while combining blinded expert rating of accuracy, quality, and safety-related content with objective readability metrics and a safety-oriented patient prompt. The present study addresses that gap. In the broader orthopedic literature, LLM performance has been examined along several distinct dimensions—patient education, adherence to clinical practice guidelines, and the management of clinical scenarios—each of which may yield different conclusions about the same model (25–27).

Accordingly, this study aimed to evaluate how accurately and completely contemporary large language models answer frequently asked postoperative questions following lumbar fusion surgery, and whether their responses include appropriate safety-related content when such content is requested. Rather than focusing solely on model superiority, we assessed clinically relevant dimensions including accuracy, response quality, safety-related content, and readability. We also aimed to identify the types of postoperative questions and clinical categories for which LLM-generated responses may remain limited, particularly those requiring individualized clinical judgment. In addition, the readability of the responses was analyzed using objective metrics (28).

## Methods

### Study design and objective

The study protocol was not registered. The six outcome domains (accuracy, composite quality, clarity, completeness, relevance, and patient safety), the readability metrics, and the two correlation analyses were specified before data collection; the category-level analysis, the count of responses meeting the operational screening criterion for potential harm, and the word count analysis were added at revision and are labeled as exploratory throughout.

This study was designed as a cross-sectional, comparative analysis to evaluate the accuracy, quality, safety-related content, and readability of responses generated by three large language model (LLM)-based artificial intelligence (AI) systems to frequently asked questions following lumbar fusion surgery. The responses were independently evaluated by three attending neurosurgeons with experience in spine surgery, who served as expert raters.

### Question selection

A total of 30 frequently asked postoperative questions were compiled based on real-world patient inquiries encountered during routine clinical practice in a neurosurgery outpatient clinic. The questions reflected the most common concerns expressed by patients during postoperative follow-up visits after lumbar fusion surgery.

Item selection was performed by three attending neurosurgeons, none of whom participated in the subsequent response evaluation (the two authors and one nonauthor colleague, M.U.E., named in the Acknowledgments), based on frequency in clinical practice and clinical relevance (29). Each surgeon independently identified the questions most frequently encountered in their own postoperative practice and drafted a candidate list of 25 questions without reference to the other surgeons' lists. The 75 nominated items were then pooled, semantically duplicate items were merged, items not specific to the postoperative period after lumbar fusion were removed, and the remaining items were ranked by how frequently they had been nominated and by the relative public search interest of their topics as indicated by Google Trends; the final 30 items were retained by consensus in a joint meeting, with disagreements resolved by discussion. To enhance content validity, questions were selected to represent a broad spectrum of postoperative concerns, including pain management, functional recovery, daily activities, and potential complications. Apart from the Google Trends check, no formal patient survey or patient-forum sampling was undertaken; the question set therefore represents the surgeons' perception of the most frequent patient inquiries, informed by search-interest data, rather than a direct sample of patient-generated questions, and this is acknowledged as a limitation of external validity (see Limitations).

All questions were formulated in clear, patient-friendly language to simulate authentic patient queries and to ensure consistency across AI model interactions. The full list of patient-oriented questions used in this study is provided in Supplementary Table 1.

### Question categories

In the question set used for response generation, the 30 questions were organized *a priori* into six clinical categories reflecting the structure of postoperative counseling: (1) activity and daily life (*n* = 10; sitting, stairs, driving, return to work, sexual activity, exercise, bending, lifting, household activities, and air travel); (2) pain and recovery (*n* = 6; leg pain, numbness, back pain, muscle weakness, recovery time, and normal vs. concerning pain); (3) wound care and hygiene (*n* = 5; suture removal, showering, swelling, redness, and discharge); (4) medications and general condition (*n* = 4; anticoagulants, antibiotics, constipation, and fever threshold); (5) complications and warning signs (*n* = 4; emergency presentation, sudden leg weakness, urinary retention, and urinary incontinence); and (6) surgical outcome (*n* = 1; recurrence). The category of each question is indicated in Supplementary Table 1. Category headings were not shown to the raters. Although the grouping itself predates response generation, the category-level analysis reported below was not part of the original analysis plan and was added at revision as an exploratory analysis; its results should be regarded as hypothesis-generating.

### Response generation

#### Artificial intelligence models

Responses were generated using three publicly available large language model-based chatbots during the predefined data collection period: ChatGPT based on GPT-5.2 (OpenAI), Gemini based on Gemini 3 Flash (Google), and Claude based on Claude Sonnet 4.6 (Anthropic). All models were accessed through their publicly available web interfaces between March 2026 and April 2026. No subscription-based, API-based, or locally modified versions were used: all three platforms were accessed through free-tier accounts, the model identifier displayed by the interface was recorded for every response, and no usage limit was reached on any platform during data collection, so no response was generated under a fallback model. The three models were of comparable tier: each was the default model served to free-tier users of its platform during the data collection period (GPT-5.2 Instant since December 2025, Gemini 3 Flash since December 2025, and Claude Sonnet 4.6 since February 2026), and none was the flagship or extended-reasoning variant of its family. This approach was selected to reflect real-world access conditions for general users seeking postoperative health information online. Because large language models are frequently updated, these model specifications should be interpreted as a time-specific snapshot of model performance.

Each question was submitted once to each model, each in a newly opened chat tab, with the chat-memory feature disabled on every platform so that no conversational context or stored information carried over between items; no custom instructions or account-level personalizations were configured. Responses were recorded immediately after generation and were not regenerated, edited, or selectively repeated. This design was chosen to simulate a real-world patient interaction with publicly available LLM interfaces. However, because LLMs are nondeterministic and can generate different responses to the same prompt across sessions or over time, a single response does not capture within-model variability; the findings should be interpreted as a snapshot of model performance during the data collection period, and repeated independent queries were not performed (see Limitations).

#### Standardized prompt

To simulate a real postoperative patient scenario and to ensure comparability across models, all questions were submitted using a single standardized prompt:

I recently underwent lumbar fusion surgery. Please answer the following question in a clear, patient-friendly, and medically accurate way. Also, if there are any warning signs or dangerous symptoms related to this question, explicitly mention them and advise when to seek medical attention.

This prompting approach ensured uniform context across all platforms and enabled evaluation of both informational accuracy and safety awareness (22). Importantly, because the prompt explicitly instructed the models to mention warning signs and to advise when to seek medical attention, the patient safety subdomain (below) measures the quality of safety-related content produced under instruction, not the models' spontaneous safety behavior. A model that would omit warning signs when asked a bare question could still score well in this design. This distinction is addressed in the Discussion and Limitations.

#### Blinding and randomization

Prior to evaluation, all 90 responses (30 questions × 3 models) were anonymized so that no source-identifying information remained, randomized in order, and reformatted into a uniform structural template (identical font, spacing and heading style; the models' own paragraph structure, lists and headings were retained, and no wording was altered). Prompt submission, response collection, coding (anonymization) and reformatting were all performed by the first author (A.K.K.), who did not take part in rating; the reformatted set was checked against the originals by the second author. The unmodified original texts were used for readability scoring (see below). Raters were blinded to the source of each response and to the study hypothesis throughout the evaluation process. In addition, raters were not involved in the generation of questions or responses, minimizing potential selection and assessment bias. None of the raters is an author of this manuscript. The code key linking each response to its model was held by the first author and withheld from the raters until all scoring was complete.

### Evaluation process

#### Raters

Three independent attending neurosurgeons with experience in spine surgery evaluated all responses. The same three raters scored every response, generating triplicate ratings for each item. None of the raters is an author of this manuscript; they are named in the Acknowledgments. Each rater scored independently, without discussion, and blinded to model identity.

#### Outcome measures

Each response was evaluated for accuracy and four quality subdomains—clarity, completeness, relevance, and patient safety—each scored on a 4-point Likert scale (1 = poor; 4 = excellent):

Accuracy was rated using a single 4-point Likert item (1 = incorrect or misleading; 2 = partially correct, with an important error or omission present; 3 = mostly correct, with an only minor imprecision; 4 = fully correct and medically appropriate) (30). Raters were instructed that accuracy should reflect medical correctness and whether any error or omission could meaningfully affect the patient's understanding or actions.

#### Reference standard for accuracy

No single guideline covers all postoperative lumbar fusion recommendations, and practice patterns vary among surgeons. No external answer key was therefore constructed; accuracy was judged by the raters, all practicing spine surgeons, against current published evidence and accepted postoperative practice (for example, established recommendations on suture removal, antibiotic prophylaxis, fever thresholds, cauda equina and neurological red flags, and thromboembolism prevention during travel), supplemented by the routine postoperative protocol of the authors' institution for topics without specific published guidance. The written scoring guide instructed raters that, for questions depending on individual recovery, surgical details, or clinician preference—such as lifting restrictions, return to exercise, sexual activity, and long-distance travel—a reasonable range or conditional answer should not be penalized solely because it does not give one fixed time point; in practice, responses lost accuracy points on these items when they stated a definitive time point outside commonly accepted practice, contained contraindicated advice, or omitted the need for individualized clearance by the treating surgeon. Disagreements between raters were not adjudicated; the three independent ratings were retained and averaged, and interrater reliability is reported. Reliance on expert judgment rather than an external reference standard is acknowledged as a limitation.

Quality was evaluated using a structured framework adapted from a modified DISCERN-based approach, including four subdomains: clarity, completeness, relevance, and patient safety. Each subdomain was rated on a four-point Likert scale (1 = poor; 4 = excellent), yielding a total composite score ranging from 4 to 16, with higher scores reflecting better overall quality. In addition to the composite score, each subdomain was also analyzed separately (31).

Rationale for the DISCERN modification. The original DISCERN instrument comprises 16 items and was designed to appraise written consumer information about treatment choices, with an emphasis on stated aims, source citation, currency of information, treatment alternatives, and shared decision-making (31). Several of these items are not applicable to a single chatbot answer to a single postoperative question: LLM responses generally do not cite verifiable sources or publication dates, do not discuss alternative treatments, and were not intended to support a treatment decision. Consistent with earlier LLM evaluations that adapted DISCERN for this reason (30), we retained the DISCERN principles most relevant to a discrete answer—clear presentation (clarity), coverage of the relevant content (completeness), and focus on the question asked (relevance)—and omitted the source, currency, treatment alternative, and shared decision-making items. Because the study addressed postoperative counseling, a fourth subdomain, patient safety, was added to capture whether a response identified warning signs and advised timely medical contact. The resulting four-item instrument is therefore a DISCERN-informed framework rather than a validated DISCERN score; it has not been validated, and its composite score should be interpreted accordingly. The full rubric with anchor descriptors is provided as Supplementary Table 2.

Clarity was defined as the ease of understanding of the response for a general patient population (1 = difficult to understand or poorly organized; 2 = understandable with effort, substantial jargon or confusing wording; 3 = generally clear, minor jargon or organizational issues; 4 = clear, well organized and easy to understand). Completeness referred to the extent to which the response adequately covered all relevant aspects of the question (1 = major relevant information missing; 2 = several important points missing; 3 = most relevant information included, minor omissions; 4 = all important aspects adequately covered). Relevance was defined as how directly the response addressed the patient's query without including unnecessary or unrelated information (1 = mostly off-topic or generic; 2 = partly relevant with considerable unnecessary information; 3 = directly relevant with minor digressions; 4 = focused and directly addresses the question). Patient safety reflected the inclusion of appropriate medical caution, such as the identification of warning signs and recommendations for seeking medical attention when necessary (1 = unsafe advice, inappropriate reassurance or important warning signs omitted; 2 = safety information incomplete or advice on seeking care unclear; 3 = key safety information included with minor omissions; 4 = appropriate warning signs and clear advice on when to seek medical care). These anchor descriptors, together with brief scoring notes, were provided to the raters as a written scoring guide before scoring (Supplementary Table 2).

#### Operational screening criterion for potential harm

Because mean Likert scores may obscure rare but clinically important failures, we additionally counted, for each model, the number and proportion of responses that met an operational screening criterion for potential harm. This criterion was defined as any response that received a score of ≤2 on accuracy (“incorrect or misleading” or “partially correct”) or on patient safety (“unsafe advice, inappropriate reassurance or important warning signs omitted” or “safety information incomplete”) from at least one rater. Meeting this criterion is a screening classification that flags a response for review because a single rater assigned a low score; it does not constitute an adjudicated determination that the response was actually harmful, and in flagged cases, the other two raters did not concur. As a secondary, more lenient indicator, we also report the number of responses whose rater-averaged patient safety score was ≤3.0. This analysis was added at revision and was not prespecified.

#### Response length

Response length was quantified as the number of words in each response, excluding the prompt and the question text, using a whitespace-/punctuation-based tokenizer applied to the response as delivered by the interface. Word count was compared across models and examined as a potential confounder of the relationship between readability and completeness.

#### Readability assessment

Readability was evaluated for each response in its original form, without modification, i.e., as delivered by the chat interface, before the visual reformatting applied for rating. Because reformatting changed only typography and not wording, sentence boundaries, or list structure, the text scored for readability and the text seen by the raters were identical in content; readability indices and expert ratings therefore describe the same response. Indices were computed with the online tool Readable (readable.com; Added Bytes Ltd, Brighton, UK), into which each response was pasted as plain text with its original line structure; the tool treats each line break, and therefore each bullet point or heading, as a sentence boundary, and numerals and symbols were retained. Three validated readability metrics were calculated for every response: the Flesch Reading Ease score, the Flesch–Kincaid Grade Level, and the Gunning Fog index. Higher Flesch Reading Ease values reflect easier text, whereas lower Flesch–Kincaid Grade and Gunning Fog values indicate texts that require lower educational levels to comprehend (28, 32, 33). Because readability formulas are less reliable for very brief texts, responses shorter than 100 words were to be flagged; no response fell below this threshold (shortest response 227 words), so no response was flagged or excluded.

### Statistical analysis

Categorical variables were summarized as frequencies and percentages; continuous variables were expressed as mean ± standard deviation, together with median and interquartile range (Q1–Q3). Because each of the three raters scored every response from every model, model-level comparisons were conducted using the mean of the three raters' scores for each item, treating the question as the unit of analysis.

Differences among the three AI models were tested using the Friedman test, given the repeated-measures structure of the data (each question answered by all three models). Pairwise comparisons were performed using the Wilcoxon signed-rank test (with normal approximation and continuity correction), and *p*-values were adjusted for multiple comparisons using the Holm correction. Effect sizes for the Friedman test were reported as Kendall's W and interpreted as small (<0.3), moderate (0.3–0.5), or large (>0.5). Where an overall Friedman test was significant, but no pairwise comparisons survived the Holm correction, the domain was reported as showing a global difference without a demonstrable pairwise superiority of any single model. Because six correlated outcomes were tested, and because the composite quality score is the arithmetic sum of the four subdomains and is therefore not independent of them, the domain-level omnibus tests are regarded as exploratory; Holm-adjusted *p*-values across the six outcome families are also reported in Table 1.

**Table 1:** Overall comparison of models based on expert scores.

Domain	Test statistic	*p* ^a^	Kendall's *W*	Holm-adjusted *p*^b^
Accuracy	*χ*^2^ = 3.694	0.158	0.062	0.158
Quality	*χ*^2^ = 12.733	0.002	0.212	0.012
Clarity	*χ*^2^ = 7.714	0.021	0.129	0.084
Completeness	*χ*^2^ = 10.638	0.005	0.177	0.025
Relevance	*χ*^2^ = 6.290	0.043	0.105	0.129
Patient safety	*χ*^2^ = 6.029	0.049	0.100	0.129

Kendall's *W*: effect size.

a Friedman test.

b Holm correction applied across the six outcome families (exploratory).

No *a priori* sample size calculation was performed; the number of questions was chosen to exceed that of comparable studies (20, 22, 29). With 30 paired observations, a paired-samples approximation indicates approximately 80% power (*α* = 0.05, two-sided) to detect a standardized paired difference of 0.53 between two models (the Wilcoxon signed-rank test retains about 95% of this efficiency); the study may therefore have been underpowered to detect small between-model differences. Statistical significance is therefore distinguished from clinical relevance throughout, with effect sizes reported alongside *p*-values.

Category-level analysis was exploratory. Within each category containing at least four questions, mean scores were compared across models with the Friedman test; because of the small number of questions per category, these tests are presented descriptively without pairwise testing or multiplicity correction and should not be used to infer model superiority within a category. Word counts were compared across models with the Friedman test and Holm-corrected Wilcoxon signed-rank tests, and the association between word count and expert ratings was examined with Spearman's rank correlation, both pooled across models and within each model.

Interrater reliability was assessed using the intraclass correlation coefficient (ICC) under a two-way random-effects, absolute-agreement model (34). Single-rater reliability was reported as ICC (2, 1), and the reliability of the mean of three raters was reported as ICC (2, 3), together with their 95% confidence intervals. ICC analyses were performed both pooled across all model responses and separately for each model.

Question-level similarity between models was examined pairwise using Spearman's rank correlation coefficient and overall (across all three models) using the ICC. Comparisons of readability metrics across the three models were performed using the Friedman test followed by Holm-corrected Wilcoxon signed-rank tests. Associations between expert ratings and readability metrics were examined with Spearman's rank correlation.

A two-sided *p*-value < 0.05 was considered statistically significant. All statistical analyses were performed using IBM SPSS Statistics version 26 (IBM Corp., Armonk, NY, USA) and the R statistical environment version 4.5.1.

### Ethical considerations

This study did not involve human participants or identifiable patient data; all responses were generated by publicly available AI systems and rated by clinicians. Therefore, formal ethics committee approval was not required.

## Results

### Descriptive findings

Overall, mean expert scores were high across all evaluated domains for all three models. Claude had numerically the highest mean values in accuracy, clarity, completeness, quality, and patient safety, whereas ChatGPT had numerically the highest relevance scores. Gemini generally yielded lower scores, especially in quality and completeness (Table 2).

**Table 2:** Descriptive statistics of expert scores by model.

Domain	Model	Mean ± SD	Median (Q1–Q3)	Min.–max.
Accuracy	ChatGPT	3.756 ± 0.289	4.000 (3.417–4.000)	3.333–4.000
	Gemini	3.689 ± 0.371	3.833 (3.333–4.000)	3.000–4.000
	Claude	3.844 ± 0.227	4.000 (3.667–4.000)	3.333–4.000
Quality	ChatGPT	15.011 ± 0.809	15.333 (14.667–15.667)	13.333–16.000
	Gemini	14.556 ± 0.729	14.500 (14.333–15.000)	12.333–16.000
	Claude	15.033 ± 0.813	15.167 (14.333–15.917)	13.333–16.000
Clarity	ChatGPT	3.700 ± 0.320	3.667 (3.667–4.000)	3.000–4.000
	Gemini	3.600 ± 0.332	3.667 (3.333–4.000)	3.000–4.000
	Claude	3.767 ± 0.292	3.833 (3.667–4.000)	3.000–4.000
Completeness	ChatGPT	3.800 ± 0.298	4.000 (3.667–4.000)	3.000–4.000
	Gemini	3.600 ± 0.365	3.667 (3.333–4.000)	3.000–4.000
	Claude	3.811 ± 0.272	4.000 (3.667–4.000)	3.333–4.000
Relevance	ChatGPT	3.744 ± 0.299	3.667 (3.667–4.000)	3.000–4.000
	Gemini	3.667 ± 0.303	3.667 (3.417–4.000)	3.000–4.000
	Claude	3.633 ± 0.320	3.667 (3.333–4.000)	3.000–4.000
Patient safety	ChatGPT	3.767 ± 0.317	4.000 (3.667–4.000)	3.000–4.000
	Gemini	3.689 ± 0.315	3.667 (3.417–4.000)	3.000–4.000
	Claude	3.822 ± 0.300	4.000 (3.667–4.000)	3.000–4.000

SD, standard deviation. Scores are the mean of three raters per response (*n* = 30 responses per model). Values were recalculated from the rater-level dataset at revision.

The distribution of accuracy scores indicated a ceiling effect, with most responses receiving high ratings across all three models: no response received a rater-averaged accuracy score below 3.0, and 207 of 270 individual accuracy ratings (76.7%) were the maximum score of 4. Therefore, differences in absolute accuracy scores between models should be interpreted cautiously.

### Comparison of models

No statistically significant difference was observed among models in terms of accuracy (Friedman *χ*^2^ = 3.694, *p* = 0.158). However, significant differences were identified for quality (*χ*^2^ = 12.733, *p* = 0.002), clarity (*χ*^2^ = 7.714, *p* = 0.021), completeness (*χ*^2^ = 10.638, *p* = 0.005), relevance (*χ*^2^ = 6.290, *p* = 0.043), and patient safety (*χ*^2^ = 6.029, *p* = 0.049). Effect sizes were small across these domains (Kendall's *W* = 0.100–0.212). When the Holm correction was additionally applied across the six outcome families, only quality (adjusted *p* = 0.012) and completeness (adjusted *p* = 0.025) remained significant; the omnibus differences in clarity (0.084), relevance (0.129), and patient safety (0.129) did not, and are therefore regarded as exploratory.

Although several model-level comparisons were statistically significant, Kendall's W values indicated small effect sizes, suggesting that the magnitude of between-model differences was limited. In absolute terms, the largest between-model difference in any subdomain mean was 0.21 points on a 4-point scale (completeness, Gemini vs. Claude), and 0.48 points on the 16-point composite scale.

Across these domains, Claude generally had numerically higher scores, whereas Gemini tended to have lower scores, particularly in quality and completeness. ChatGPT showed comparable performance to Claude in most domains and the numerically highest scores in relevance (Table 2). The distribution of expert scores across models is illustrated in Figure 1.

**Figure 1:**
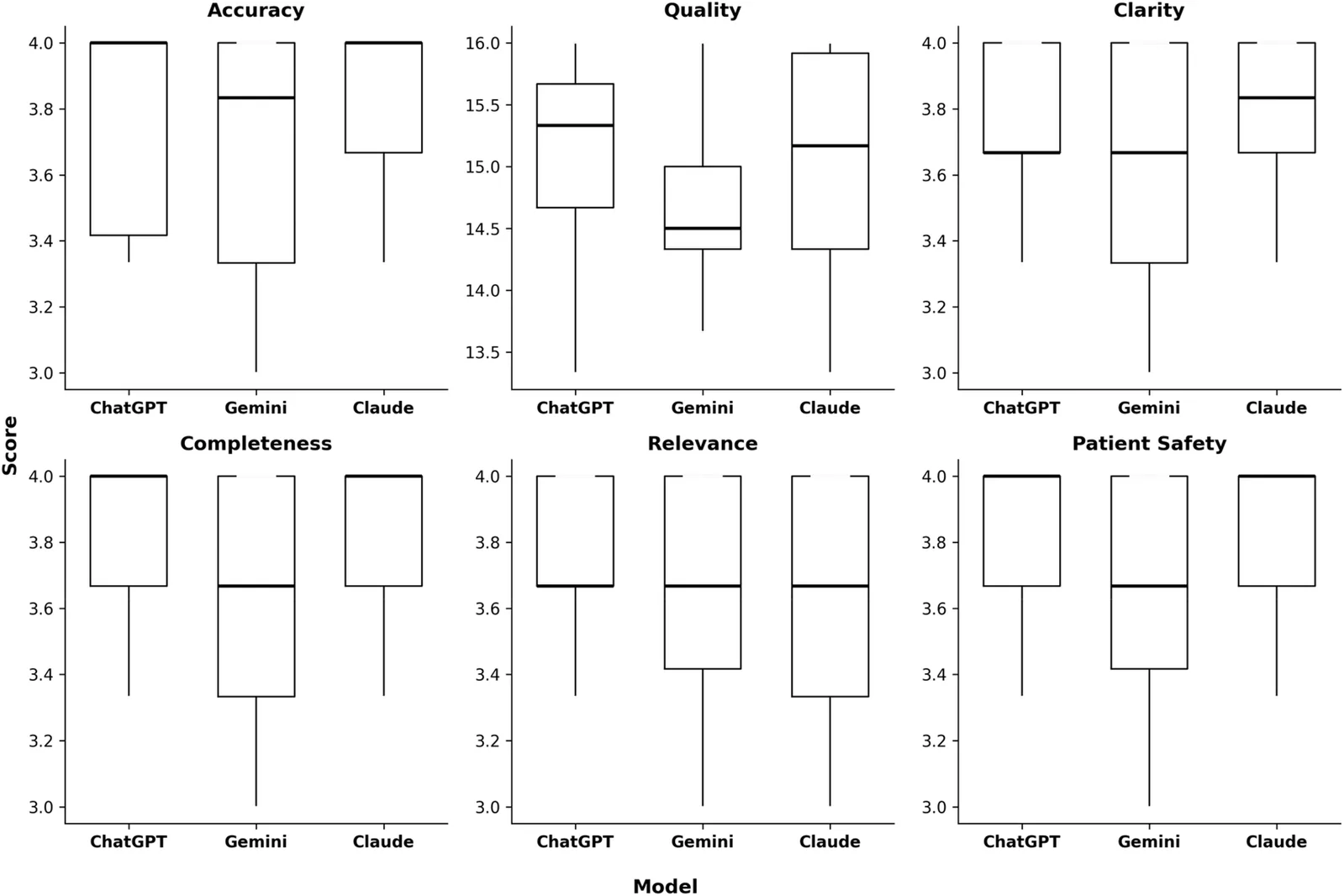
Distribution of expert scores of AI models across the evaluated dimensions.

### Pairwise comparisons

Pairwise analyses revealed that Gemini scored significantly lower than both ChatGPT and Claude on quality and completeness. No significant differences were observed between ChatGPT and Claude in these domains.

For clarity, Gemini scored lower than Claude after Holm correction (adjusted *p* = 0.037), with no other significant pairwise difference. Although overall tests were significant, pairwise comparisons were not significant after Holm correction; consequently, no individual model can be regarded as superior to another in these two domains based on the present data. No significant pairwise differences were observed for accuracy (Table 3).

**Table 3:** Pairwise comparisons of expert scores across models.

Domain	Comparison	Test statistic	*p* ^a^	Adjusted *p*-value
Accuracy	ChatGPT—Gemini	*W* = 92.000	0.634	0.634
	ChatGPT—Claude	*W* = 27.000	0.196	0.391
	Gemini—Claude	*W* = 32.500	0.035	0.105
Quality	ChatGPT—Gemini	*W* = 65.500	0.009	0.018
	ChatGPT—Claude	*W* = 141.000	0.807	0.807
	Gemini—Claude	*W* = 27.000	<0.001	0.001
Clarity	ChatGPT—Gemini	*W* = 91.000	0.245	0.491
	ChatGPT—Claude	*W* = 70.500	0.521	0.521
	Gemini—Claude	*W* = 29.500	0.012	0.037
Completeness	ChatGPT—Gemini	*W* = 19.000	0.003	0.009
	ChatGPT—Claude	*W* = 59.000	0.977	0.977
	Gemini—Claude	*W* = 15.000	0.006	0.012
Relevance	ChatGPT—Gemini	*W* = 29.000	0.140	0.280
	ChatGPT—Claude	*W* = 24.500	0.018	0.053
	Gemini—Claude	*W* = 56.000	0.318	0.318
Patient safety	ChatGPT—Gemini	*W* = 37.500	0.190	0.379
	ChatGPT—Claude	*W* = 51.000	0.362	0.379
	Gemini—Claude	*W* = 34.500	0.040	0.120

a Wilcoxon signed-rank test.

Adjusted *p*: Holm-corrected *p*-value.

### Performance by clinical category

Table 4 presents rater-averaged accuracy, composite quality, and patient safety scores by clinical category. Pooled across models, the highest scores were observed for medications and general condition (mean accuracy 3.92, quality 15.44, safety 3.94) and the lowest composite quality scores were observed for the single surgical-outcome question on recurrence (13.67) and for pain and recovery (14.69) and activity and daily life (14.78). Patient safety scores were lowest in the wound-care and hygiene category (3.56), driven by the questions on showering (Q18) and wound discharge (Q21), each of which received a rater-averaged safety score of 3.00 from all three models.

**Table 4:** Expert scores by clinical category and model (exploratory analysis) (rater-averaged, mean ± SD).

Category (*n* questions)	Domain	ChatGPT	Gemini	Claude	*p* ^a^
Activity and daily life (*n* = 10)	Accuracy	3.73 ± 0.31	3.50 ± 0.39	3.87 ± 0.17	0.034
	Quality	14.60 ± 0.68	14.63 ± 0.46	15.10 ± 0.55	0.063
	Patient safety	3.73 ± 0.26	3.73 ± 0.31	3.87 ± 0.23	0.185
Pain and recovery (*n* = 6)	Accuracy	3.89 ± 0.27	3.72 ± 0.39	3.89 ± 0.27	0.584
	Quality	15.39 ± 0.25	14.17 ± 1.05	14.50 ± 0.94	0.022
	Patient safety	3.89 ± 0.17	3.67 ± 0.21	3.78 ± 0.27	0.291
Wound and hygiene (*n* = 5)	Accuracy	3.80 ± 0.30	3.73 ± 0.43	3.73 ± 0.28	0.717
	Quality	14.93 ± 1.19	14.60 ± 0.37	15.20 ± 1.10	0.513
	Patient safety	3.60 ± 0.55	3.47 ± 0.45	3.60 ± 0.55	0.135
Medications and general condition (*n* = 4)	Accuracy	3.75 ± 0.32	4.00 ± 0.00	4.00 ± 0.00	0.135
	Quality	15.67 ± 0.47	15.00 ± 0.98	15.67 ± 0.67	0.150
	Patient safety	4.00 ± 0.00	3.83 ± 0.33	4.00 ± 0.00	0.368
Complications and warning signs (*n* = 4)	Accuracy	3.67 ± 0.27	3.67 ± 0.27	3.67 ± 0.27	1.000
	Quality	15.33 ± 0.47	14.75 ± 0.63	15.00 ± 0.82	0.146
	Patient safety	3.75 ± 0.32	3.83 ± 0.19	3.83 ± 0.19	0.779
Surgical outcome (*n* = 1)	Accuracy	3.33	4.00	4.00	—
	Quality	13.33	13.33	14.33	—
	Patient safety	3.33	3.33	4.00	—

a Friedman test within category, exploratory; not computed for the single-question category. Quality = composite of clarity, completeness, relevance, and patient safety (range 4–16). SD not defined for *n* = 1.

Exploratory within-category comparisons showed a significant global difference among models only for accuracy in the activity and daily life category (Friedman *p* = 0.034; Gemini lowest, 3.50 vs. 3.73 for ChatGPT and 3.87 for Claude) and for composite quality in the pain and recovery category (*p* = 0.022; ChatGPT highest, 15.39 vs. 14.17 for Gemini and 14.50 for Claude). No other within-category difference reached significance, and given the small number of questions per category (4–10) these findings are hypothesis-generating only. Notably, the overall advantage of ChatGPT and Claude over Gemini in composite quality was not uniform: in the activity and daily life category, Gemini and ChatGPT were indistinguishable (14.63 vs. 14.60), whereas the gap was largest for pain and recovery.

### Operational screening criterion for potential harm

No response from any model received the lowest score (1) on any domain from any rater, and no rater-averaged accuracy or patient safety score fell below 3.0. Applying the operational definition described in the Methods (a score of ≤2 on accuracy or patient safety from at least one rater), 0 of 30 ChatGPT responses (0%), 0 of 30 Claude responses (0%), and 2 of 30 Gemini responses (6.7%) met the operational screening criterion for potential harm (Table 5). Both Gemini responses concerned wound care: the response on when showering is permitted (Q18) received a patient safety score of 2 from one rater, and the response on wound discharge (Q21) received scores of 2 for both accuracy and patient safety from the same rater. In both cases, the other two raters scored the response 3 or 4, so the rater-averaged scores remained ≥3.0. Under the more lenient indicator (rater-averaged patient safety ≤ 3.0), the same two questions were flagged for all three models (Q18 and Q21; 2 of 30 responses per model), indicating that the safety concern in these items was question-related rather than model-specific. No response failed to mention any warning sign, gave inappropriate reassurance for a red-flag symptom (fever, new neurological deficit, bladder or bowel dysfunction, wound discharge), or omitted a recommendation to seek medical attention where one was warranted.

**Table 5:** Number and proportion of responses meeting the operational screening criterion for potential harm, by model (*n* = 30 responses per model).

Indicator	ChatGPT, *n* (%)	Gemini, *n* (%)	Claude, *n* (%)
Any rater score of 1 on any domain	0 (0)	0 (0)	0 (0)
Accuracy ≤ 2 from ≥1 rater	0 (0)	1 (3.3)	0 (0)
Patient safety ≤ 2 from ≥1 rater	0 (0)	2 (6.7)	0 (0)
Screen-positive for potential harm (either of the above)	**0** **(0)**	**2** **(6.7)**	**0** **(0)**
Rater-averaged patient safety ≤ 3.0	2 (6.7)	2 (6.7)	2 (6.7)
Rater-averaged accuracy ≤ 3.0	0 (0)	4 (13.3)	0 (0)

Rater-level indicators refer to individual ratings (90 per model) summarized at the response level; the rater-averaged patient safety ≤ 3.0 responses were Q18 (showering) and Q21 (wound discharge) for every model.

Bold values indicate the primary screening indicator (screen-positive for potential harm, i.e., either an accuracy score ≤2 or a patient safety score ≤2 from at least one rater).

### Inter-rater reliability

Interrater reliability was low to moderate at the single-rater level but improved when mean scores were used. The highest agreement was observed for quality [ICC(2, 3) = 0.768], whereas relevance demonstrated the lowest agreement [ICC(2, 1) = 0.154; ICC(2, 3) = 0.353]. Single-rater ICC values were below 0.50 for every domain except quality, and even the three-rater averages remained below 0.50 for clarity, relevance, and patient safety, indicating that a substantial share of the variance in these subdomain scores reflects rater disagreement rather than differences between responses. These findings support the use of averaged ratings across evaluators for the composite quality score and, more tentatively, for completeness and accuracy; for clarity, relevance and patient safety, where reliability remained poor by the criteria of Koo and Li (34), between-model findings should be regarded as provisional (Table 6).

**Table 6:** Inter-rater reliability for all model responses.

Domain	ICC(2, 1)	95% CI	*p*	ICC(2, 3)	95% CI	*p*
Accuracy	0.250	0.120–0.390	<0.001	0.500	0.300–0.650	<0.001
Quality	0.524	0.370–0.650	<0.001	0.768	0.640–0.850	<0.001
Clarity	0.211	0.080–0.350	<0.001	0.445	0.210–0.620	<0.001
Completeness	0.324	0.200–0.460	<0.001	0.590	0.420–0.720	<0.001
Relevance	0.154	0.030–0.290	0.007	0.353	0.080–0.550	0.007
Patient safety	0.246	0.120–0.380	<0.001	0.494	0.290–0.650	<0.001

ICC: A two-way random-effects model with absolute agreement was used. ICC (2, 1) represents a single rater, while ICC (2, 3) reflects the average of three raters.

### Model similarity

Question-level similarity analysis revealed moderate correlations between models in several domains (Table 7). The strongest correlations were observed for quality scores, particularly between Gemini and Claude (Spearman *ρ* = 0.690, *p* < 0.001; ChatGPT–Claude *ρ* = 0.570, *p* = 0.001; ChatGPT–Gemini *ρ* = 0.425, *p* = 0.019), with an across-model ICC of 0.452 (95% CI: 0.23–0.66). Relevance also showed consistent moderate agreement between all pairs (*ρ* = 0.56–0.59; ICC: 0.500).

**Table 7:** Question-level similarity of expert scores between models.

Domain	ChatGPT–Gemini *ρ* (*p*)	ChatGPT–Claude *ρ* (*p*)	Gemini–Claude *ρ* (*p*)	ICC across models (95% CI)
Accuracy	0.072 (0.705)	0.154 (0.417)	0.381 (0.038)	0.164 (−0.04–0.41)
Quality	0.425 (0.019)	0.570 (0.001)	0.690 (<0.001)	0.452 (0.23–0.66)
Clarity	−0.011 (0.953)	0.076 (0.692)	0.510 (0.004)	0.166 (−0.03–0.41)
Completeness	0.405 (0.026)	0.233 (0.216)	0.492 (0.006)	0.309 (0.10–0.54)
Relevance	0.586 (<0.001)	0.562 (0.001)	0.586 (<0.001)	0.500 (0.29–0.69)
Patient safety	0.352 (0.057)	0.289 (0.121)	0.277 (0.138)	0.479 (0.26–0.68)

*ρ*, Spearman's rank correlation between the rater-averaged scores of two models across the 30 questions; ICC, two-way random-effects, absolute-agreement, single-measure ICC treating the three models as “raters” of each question.

In contrast, similarity was low for accuracy and clarity (across-model ICC: 0.164 and 0.166; only the Gemini–Claude pair correlated significantly, *ρ* = 0.381 and 0.510), indicating that models did not consistently produce similar responses across these domains; that is, a question that one model answered well was not necessarily answered well by another.

### Response length

Response length differed markedly among models (Friedman *χ*^2^ = 36.47, *p* < 0.001; Table 8). ChatGPT responses were shortest (mean 294 ± 64 words; median 278), whereas Claude (462 ± 65; median 477) and Gemini (445 ± 33; median 448) produced responses of similar length. Holm-corrected pairwise comparisons confirmed that ChatGPT responses were shorter than those of both Claude and Gemini (both adjusted *p* < 0.001), with no difference between Claude and Gemini (*p* = 0.165).

**Table 8:** Response length (word count) by model and its association with expert ratings and readability metrics.

Variable	ChatGPT	Gemini	Claude	*p*
Words, mean ± SD	294 ± 64	445 ± 33	462 ± 65	<0.001^a^
Words, median (Q1–Q3)	278 (259–309)	448 (421–466)	477 (432–508)	
Words, min–max	227–582	389–508	278–569	
Pairwise, Holm-adjusted p	vs. Gemini <0.001; vs. Claude <0.001	vs. Claude 0.165		
*ρ*, words vs. completeness (*p*)	−0.24 (0.196)	−0.15 (0.420)	−0.17 (0.383)	pooled −0.22 (0.039)
*ρ*, words vs. quality (*p*)	−0.22 (0.238)	0.19 (0.319)	−0.43 (0.019)	pooled −0.24 (0.026)
*ρ*, words vs. accuracy (*p*)				pooled 0.02 (0.833)
*ρ*, words vs. patient safety (*p*)				pooled −0.03 (0.753)
*ρ*, words vs. Flesch–Kincaid Grade (*p*)	0.07 (0.707)	−0.02 (0.901)	0.10 (0.603)	pooled 0.01 (0.922)
*ρ*, words vs. Flesch Reading Ease (*p*)	−0.04 (0.820)	0.17 (0.357)	−0.06 (0.772)	pooled 0.07 (0.505)
*ρ*, words vs. Gunning Fog (*p*)	0.05 (0.775)	−0.18 (0.343)	0.10 (0.596)	pooled −0.05 (0.660)

a Friedman test; pairwise: Holm-corrected Wilcoxon signed-rank tests; *ρ*: Spearman's rank correlation. Word count excludes the prompt and question text.

Pooled across the 90 responses, word count was not positively associated with expert ratings. It showed weak negative correlations with composite quality (Spearman *ρ* = −0.24, *p* = 0.026), completeness (*ρ* = −0.22, *p* = 0.039), and relevance (*ρ* = −0.24, *p* = 0.025), and no association with accuracy (*ρ* = 0.02, *p* = 0.83), clarity (*ρ* = −0.07, *p* = 0.53), or patient safety (*ρ* = −0.03, *p* = 0.75). Within models, the only significant association was a negative correlation between length and composite quality for Claude (*ρ* = −0.43, *p* = 0.019); the correlation between length and completeness was non-significant and negative in direction for all three models. Thus longer responses were not rated as more complete, and the lower completeness of Gemini could not be attributed to shorter output, as its responses were of comparable length to Claude's and substantially longer than ChatGPT's. Word count was likewise unrelated to readability: pooled Spearman correlations between word count and the Flesch–Kincaid Grade Level, Flesch Reading Ease, and Gunning Fog index were 0.01 (*p* = 0.922), 0.07 (*p* = 0.505), and −0.05 (*p* = 0.660), respectively, and no within-model correlation exceeded |*ρ*| = 0.18 (all *p* > 0.3; Table 8). Readability metrics were also unrelated to completeness at the response level (Flesch–Kincaid *ρ* = −0.07, Flesch Reading Ease *ρ* = 0.01, Gunning Fog *ρ* = 0.02; all *p* > 0.5).

### Readability analysis

Significant differences in readability were observed among the models. Gemini demonstrated the most favorable readability profile, with lower Flesch–Kincaid Grade Level and Gunning Fog Index values, and higher Flesch Reading Ease scores compared with ChatGPT and Claude (all *p* < 0.05).

ChatGPT and Claude showed similar readability characteristics (Table 9) despite a difference of nearly 170 words in mean length, and Gemini was more readable than Claude at a comparable length; readability differences between models were therefore not a function of response length. The boxplot comparison shows that Gemini consistently demonstrates better readability (lower Flesch–Kincaid Grade and Gunning Fog, higher Flesch Reading Ease), whereas ChatGPT and Claude tend to produce more complex texts with higher reading level requirements (Figure 2).

**Table 9:** Descriptive statistics of readability metrics by model.

Readability Metric	Model	Mean ± SD	Median (Q1–Q3)	Min–Max
Flesch–Kincaid Grade	ChatGPT	7.140 ± 1.166	7.050 (6.225–7.875)	5.300–9.300
	Gemini	6.753 ± 0.863	6.700 (6.100–7.275)	5.500–8.500
	Claude	7.190 ± 0.832	7.100 (6.650–7.800)	5.800–8.800
Flesch reading ease	ChatGPT	56.520 ± 8.004	56.300 (49.850–61.400)	40.300–70.100
	Gemini	61.387 ± 6.560	60.400 (57.350–66.025)	48.400–71.000
	Claude	55.627 ± 6.742	57.100 (50.225–59.850)	43.100–66.900
Gunning fog	ChatGPT	8.960 ± 1.338	8.800 (8.150–10.100)	7.100–11.500
	Gemini	8.287 ± 0.894	8.400 (7.650–8.775)	6.900–10.300
	Claude	8.967 ± 0.913	8.850 (8.325–9.375)	7.400–11.100

SD, standard deviation.

**Figure 2:**
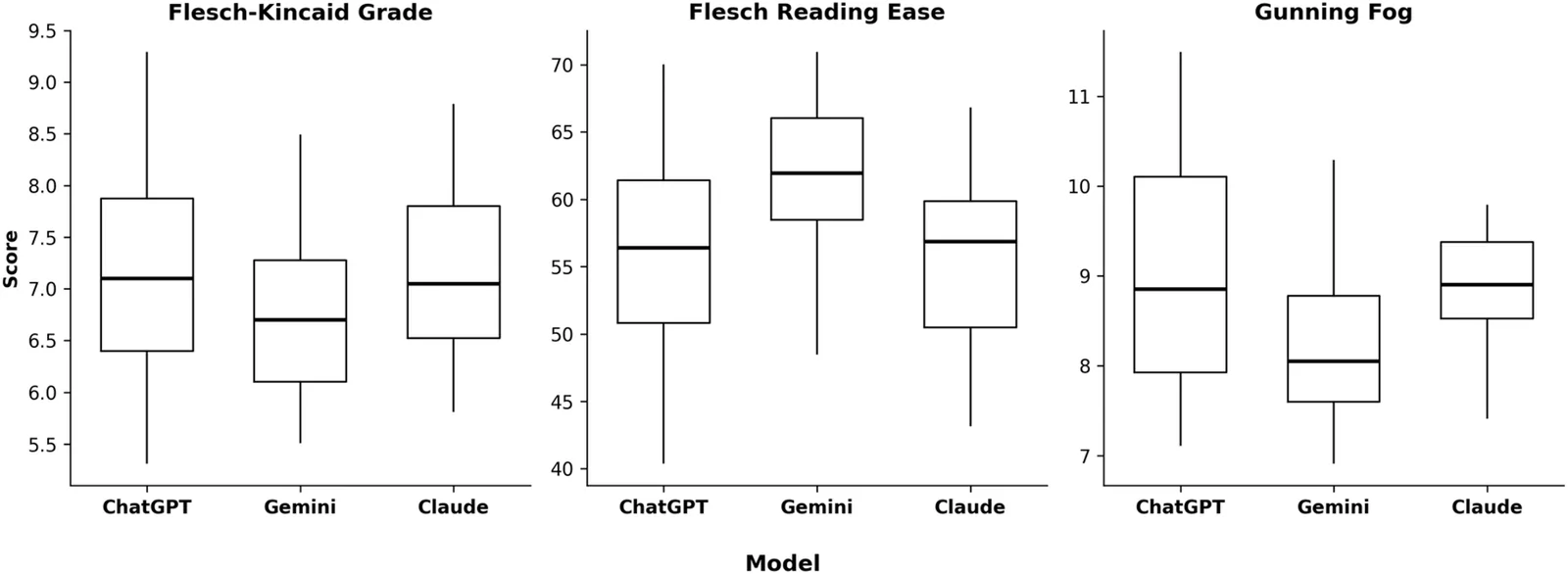
Distribution of readability metrics across AI models.

### Association between expert ratings and readability

At the level of individual responses (*n* = 90), expert ratings were essentially unrelated to readability (Table 10). Completeness in particular showed no association with any readability metric (Flesch–Kincaid *ρ* = −0.07, *p* = 0.516; Flesch Reading Ease *ρ* = 0.01, *p* = 0.948; Gunning Fog *ρ* = 0.02, *p* = 0.882). The only correlation reaching significance was a weak positive association between clarity ratings and the Gunning Fog index (*ρ* = 0.22, *p* = 0.038), i.e., responses rated as clearer by surgeons tended to have slightly higher, not lower, reading-level scores. The inverse readability-completeness pattern described above is therefore a between-model pattern—Gemini was, on average, both more readable and less complete—rather than a within-response relationship.

**Table 10:** Spearman correlations between rater-averaged expert scores and readability metrics across the 90 responses.

Expert rating domain	Flesch–Kincaid grade *ρ* (*p*)	Flesch reading ease *ρ* (*p*)	Gunning fog *ρ* (*p*)
Accuracy	0.02 (0.817)	−0.06 (0.600)	0.03 (0.759)
Quality	0.10 (0.345)	−0.18 (0.083)	0.17 (0.112)
Clarity	0.15 (0.152)	−0.18 (0.098)	0.22 (0.038)
Completeness	−0.07 (0.516)	0.01 (0.948)	0.02 (0.882)
Relevance	0.03 (0.755)	−0.10 (0.342)	0.05 (0.656)
Patient safety	0.12 (0.275)	−0.17 (0.114)	0.16 (0.134)

Positive *ρ* with Flesch–Kincaid Grade or Gunning Fog indicates that higher-rated responses required a higher reading level; positive *ρ* with Flesch Reading Ease indicates the opposite.

Against the sixth-grade benchmark recommended for patient materials (35), 7 of 30 Gemini responses (23.3%), 3 of 30 ChatGPT responses (10.0%), and 2 of 30 Claude responses (6.7%) had a Flesch–Kincaid Grade Level of 6.0 or below (12 of 90 overall, 13.3%); 21, 13, and 13 responses, respectively, were below grade 7.

## Discussion

### Principal findings

In this study, we aimed to systematically evaluate and compare the performance of three contemporary large language models—ChatGPT, Gemini, and Claude—by analyzing their responses to 30 frequently asked questions from patients following lumbar fusion surgery. The responses were independently assessed in a blinded manner by three experienced spine surgeons.

All three models achieved consistently high mean accuracy scores, and no statistically significant differences were observed among them in this domain (20). However, the models diverged modestly in terms of response quality, completeness, and readability. Specifically, Claude tended to achieve numerically higher scores across overall quality measures, ChatGPT appeared to perform better in terms of relevance, whereas Gemini showed a more favorable profile with regard to readability (24); however, after correction across the six outcome families, robust between-model differences remained only for quality and completeness, on which Gemini scored lower than both other models. The lower clarity of Gemini relative to Claude emerged only in within-domain pairwise testing and should be regarded as exploratory, and no model could be shown to be superior to another in relevance or patient safety. Gemini scored significantly lower than both ChatGPT and Claude in terms of quality and completeness, yet exhibited the most favorable profile across readability metrics. This finding points to a potential divergence between the depth of information provided and overall readability, observed between models rather than within individual responses. The word-count analysis indicates that it is not simply a matter of response length. In exploratory, hypothesis-generating question- and category-level analyses, the models tended to receive consistently high ratings on standard, guideline-based questions, whereas apparent limitations were more common in questions involving activity timing, individualized recommendations, and interpretation of wound-related symptoms; because these analyses were exploratory, the pattern should be regarded as hypothesis-generating rather than confirmatory.

It should be emphasized at the outset that the effect sizes for all significant between-model differences were small (Kendall's *W*: 0.100–0.212), that the largest difference between any two models in any subdomain mean was 0.2 points on a 4-point scale, and that with 30 questions the study was powered only to detect moderate pairwise differences. Statistical significance in this setting should therefore not be equated with clinical significance: from the perspective of a patient reading a single answer, the three models were far more alike than different, and the clinically relevant distinctions emerged at the level of individual questions rather than models.

Our deliberate focus on the postoperative period reflects a clinically driven choice. Decisions regarding surgical management and operative technique are generally established through face-to-face discussions between patients and their physicians, guided by the trust inherent in the patient–physician relationship. Although patients may seek input from large language models during this process, final decisions are typically made together with their surgeons (13). In contrast, the postoperative period follows a different set of dynamics. Following discharge, patients resume their daily routines, yet they may not have immediate access to their physicians—especially when questions arise about mobilization, wound care, medication management, return to activity, or concerns that occur at night or on weekends. This gap may drive patients toward immediately accessible resources, such as AI-based systems (9). Thus, evaluating how large language models perform in postoperative patient queries is not solely a matter of academic interest, but one that directly relates to patient safety (15).

### Comparison with previous studies

The ability of AI-based language models to respond to patient questions in spine surgery has attracted increasing research interest in recent years. Lang et al. assessed 10 frequently asked questions on lumbar fusion surgery using ChatGPT-3.5 and Google Bard and reported that nearly 97% of responses were rated as acceptable or excellent, with no statistically significant difference between the two models (20). In a study by Hernandez et al., 19 patient questions on adult spinal deformity surgery were assessed using ChatGPT alone, demonstrating a similarly high, albeit variable, level of accuracy (22). Mejia et al. compared ChatGPT's responses regarding the management of lumbar disc herniation with established clinical guidelines, concluding that the overall level of information was satisfactory, although the model occasionally included misleading or fabricated references (21).

These findings should also be read against the rapidly expanding orthopedic literature, in which LLM performance has been examined along three distinct dimensions that are not interchangeable. The first is patient education: Karagöz et al. compared ChatGPT-5 and DeepSeek V3 as sources of patient information across foot and ankle disorders, evaluating the quality and readability of responses to lay questions (25)—the dimension addressed by the present study. The second is guideline adherence: Keçeci and Karagöz tested whether ChatGPT-4o and DeepSeek reproduce the American Academy of Orthopaedic Surgeons recommendations for clavicle fracture management, a task with an explicit reference standard (26). The third is clinical scenario management: Bayrak et al. evaluated ChatGPT, Claude, and Perplexity in a septic arthritis scenario, in which the model must reason through a time-critical clinical problem rather than inform a patient (27). Our postoperative question set sits primarily in the first dimension but borders on the third for the complications and warning-signs category, where the “patient” is effectively asking the model to triage. That a model performs well in one dimension does not imply that it performs well in another, and comparisons across studies should be made within the same dimension. The consistent picture across these studies—including ours—is that current models receive high expert ratings on standardized, guideline-like questions, whereas performance becomes less predictable as the task moves from information provision toward individualized clinical reasoning.

This study extends this body of literature in several important ways. First, whereas prior studies have largely focused on a single model or comparisons between two models, our study simultaneously evaluates three contemporary large language models with distinct architectures—ChatGPT, Gemini, and Claude. To our knowledge, Claude has not previously been evaluated on postoperative spine surgery questions, although it has been compared with ChatGPT and Gemini on medical examination items (24) and with ChatGPT and Perplexity in an orthopedic clinical scenario (27). Second, our study incorporates a larger number of questions (*n* = 30) and a more comprehensive evaluation framework, including accuracy and multiple quality domains—clarity, completeness, relevance, and patient safety—together with three separate readability metrics, response length, a count of responses meeting the operational screening criterion for potential harm, and an exploratory analysis by clinical category. Third, interrater reliability was quantified and reported for every domain rather than assumed. Agreement was good only for the composite quality score [ICC(2, 3) = 0.768] and was limited for the individual subdomains, a pattern also observed in previous studies (13); the implications are discussed below (34).

### Clinical and patient-safety implications

The category-level analysis adds a clinically interpretable layer to the model-level results. All three models scored highest on medications and general condition and on complications and warning signs—categories in which the correct content is largely codified (anticoagulant resumption, antibiotic prophylaxis, fever thresholds, cauda equina symptoms)—and lowest on the single recurrence question and on the pain-and-recovery and activity categories, in which expected timelines depend on the individual patient. The between-model differences were also category-dependent: Gemini's lower composite quality was concentrated in the pain-and-recovery category, whereas in activity and daily life it was indistinguishable from ChatGPT. Wound care and hygiene stood out as the category with the lowest safety scores for all three models, and the two questions responsible (showering and wound discharge) were also the only items flagged by the operational safety-screening indicators. These within-category observations come from an analysis added at revision and rest on four to ten questions each, and they require confirmation in larger question sets, but they suggest that model choice matters less than question type in determining the quality of the answer a patient receives.

A question-level analysis demonstrated that all three models exhibited high and consistent performance on queries involving standardized, guideline-based postoperative information. Across questions addressing return to driving, timing of return to work, suture removal, the need for antibiotic therapy, the fever threshold considered clinically concerning, and whether postoperative swelling at the surgical site should be regarded as normal, all three models consistently achieved very high scores across all evaluators. These questions typically correspond to domains in which well-established clinical recommendations exist and can be addressed using defined time intervals, threshold values, or straightforward yes/no responses. In this context, large language models with distinct architectures appeared to converge on similar, high-quality answers for such standardized questions. Because these observations derive from exploratory question-level inspection, they should be regarded as hypothesis-generating.

When the lower-performing questions were examined in detail, it became evident that a substantial proportion of them shared a common underlying characteristic. Providing an accurate answer requires access to patient-specific clinical information—such as the surgical approach, the level of fusion, the instrumentation used, accompanying comorbidities, and the individual rate of recovery. Questions such as when to begin lifting heavy objects, return to exercise, resume sexual activity, and undertake long-distance travel do not have a single “correct” answer; rather, they are shaped by the surgeon's individual recommendations and the patient's specific clinical condition. For these items, accuracy was scored against the criteria described in the Methods (a time frame within the range of published recommendations, absence of contraindicated advice, and explicit deferral to the treating surgeon), and lower scores generally reflected definitive time points stated without qualification rather than factually wrong statements. Similarly, questions such as what to do in the presence of wound discharge or when to seek emergency care require integrating the patient's current clinical status, accompanying symptoms, and individual risk factors to reach a definitive decision. Large language models can effectively reproduce commonly encountered and broadly agreed-upon information from their training data; however, given their inability to substitute for individualized clinical decision-making, their responses in such contexts are often generalized and contingent on varying conditions. A similar pattern was noted by Lang et al., who found that both ChatGPT and Bard performed worst on questions requiring individualized clinical context, such as decisions regarding surgical approach and anticipated surgical success rates (20, 21). Overall, the results indicate that large language models may support the delivery of standard, guideline-based information; however, they remain insufficient substitutes for physician judgment in scenarios requiring individualized clinical interpretation, consistent with the results of our study.

The patient safety findings suggest that all three models show a broadly consistent ability to recognize warning signs and guide patients to seek appropriate medical care when explicitly asked to do so. Overall mean scores were high and similar across models, and none of the pairwise differences remained statistically significant after Holm correction. Key complications such as fever, wound discharge, acute neurological deficits, cauda equina syndrome, deep vein thrombosis, and cerebrospinal fluid leakage were consistently identified as “red flags.” The responses generally directed patients toward urgent medical evaluation. Particularly in situations involving red flags and requiring urgent medical attention, referring patients to the hospital rather than providing a detailed interpretation was considered favorable in terms of patient safety. The cautious, safety-oriented content produced by all three models may be useful in supporting postoperative patient education under explicit safety-oriented prompting (18), but should not be interpreted as evidence of clinical safety.

Mean safety scores, however, can conceal rare but consequential failures, and we therefore also applied an operational screening criterion for potential harm (a low score from any single rater), which flags responses for review rather than adjudicating actual harm. The result is reassuring but not unqualified. No response received the lowest score on any domain, and none omitted a recommendation to seek care where one was warranted; only two of 90 responses—both from Gemini, both concerning wound care—were rated ≤2 on accuracy or safety by one of the three raters, and in both cases the other raters disagreed. The same two questions (showering and wound discharge) produced the lowest average safety scores across all three models, which points to a shared difficulty with wound-related advice, where the appropriate response depends on dressing type, surgeon-specific protocols, and the character of any drainage, rather than to a model-specific deficiency. Two caveats apply. First, these counts were obtained under a prompt that explicitly requested warning signs; the frequency of harmful omissions in unprompted use may differ and was not assessed. Second, the operational definition was applied *post hoc* to existing ratings; a prospective definition with independent adjudication of every flagged response would be preferable and is recommended for future work.

The prompt design deserves separate comment. The standardized prompt instructed the models to mention warning signs and to advise when to seek medical attention, and the patient safety subdomain then rewarded exactly this content. The high and uniform safety scores therefore demonstrate that all three models comply well with an explicit safety instruction; they do not demonstrate that the models volunteer such information spontaneously. We chose this design deliberately, because it isolates the quality of safety-related content from the question of whether a model chooses to provide it, and because it reflects how a cautious patient might phrase a query; however, a real patient asking “When can I shower?” without such an instruction may receive a materially different answer. Studies that compare prompted and unprompted safety behavior are needed before conclusions about the safety of LLMs in unsupervised patient use can be drawn, and for this reason we have avoided describing our findings as evidence that these models are “safe.”

### Readability and response length

One of the most salient findings of our study is the inverse relationship between readability and completeness across the evaluated models, which we emphasize is a pattern between models and not within responses: at the level of individual responses, readability metrics were uncorrelated with completeness (|*ρ*| ≤ 0.07; Table 10). Although the Gemini model exhibited the most advantageous performance across all three readability metrics (Flesch–Kincaid Grade, Flesch Reading Ease, and Gunning Fog), it yielded significantly lower quality and completeness scores than both ChatGPT and Claude. By comparison, ChatGPT and Claude produced more comprehensive outputs, though these were associated with a higher required reading level. A natural objection is that this pattern merely reflects response length—that shorter texts are both easier to read and less complete. The word-count analysis does not support this explanation. ChatGPT produced by far the shortest responses (mean 294 words) yet matched Claude (462 words) in completeness and quality, whereas Gemini produced responses as long as Claude's (445 words) but with lower completeness and better readability. Across all 90 responses, length was not positively correlated with completeness; if anything, the association was weakly negative. Nor was length correlated with any of the three readability metrics (all |*ρ*| ≤ 0.07 pooled), so response length cannot account for either side of the readability–completeness relationship. The observed readability advantage of Gemini was not explained by response length and may reflect a combination of linguistic complexity and model-specific formatting conventions (see Limitations); its lower completeness reflects what was omitted rather than how much was written. This model-level pattern suggests a possible tension between informational depth and readability. Texts written in shorter and simpler language are more easily understood by patients but may fail to adequately convey clinical nuance and necessary detail. In contrast, more comprehensive responses, although richer in information, may exceed the patient's reading level. As an important contextual observation, only 13.3% of responses met the sixth grade reading level recommended for patient materials (35) (Gemini 23.3%, ChatGPT 10.0%, and Claude 6.7%), and most responses fell between the seventh and ninth grades. These results suggest that none of the large language models employed for patient education independently achieves the ideal readability level, highlighting the ongoing need for physician-mediated simplification (36).

### Interrater reliability

The interrater reliability results warrant a more prominent place in the interpretation than is usual in this literature. Single-rater ICC values were poor (<0.50) for accuracy, clarity, completeness, relevance, and patient safety, and were only moderate for the composite quality score; averaging across three raters raised reliability to moderate for accuracy, completeness, and quality but left clarity, relevance, and patient safety below 0.50. This means that, for the subdomains, much of the variation in scores between responses reflects differences in how the three surgeons applied the scale rather than differences in the responses themselves. Two features of the design probably contributed: the ceiling effect, which compresses scores into the upper two points of a 4-point scale and makes the ICC highly sensitive to occasional disagreements; and the inherent subjectivity in judging relevance and safety of postoperative advice on which practice patterns vary. The practical consequence is that model-level comparisons based on averaged scores are defensible, whereas conclusions about individual subdomains—particularly relevance and patient safety—should be regarded as provisional, and the small differences reported for these subdomains should not be over-interpreted.

## Limitations

This study has several limitations. First, responses were obtained using a single prompt and a single query per question and model, each in a separate chat; given that large language models are nondeterministic and routinely generate different responses to the same question over time, a single response cannot be assumed to represent a model's typical performance, and within-model consistency was not assessed. Small between-model differences of the magnitude observed here could plausibly be reversed on repeated querying. Future studies should submit each question several times, independently, and report within-model variability alongside between-model differences. Second, the 30 questions were compiled by neurosurgeons on the basis of their clinical experience rather than sampled directly from patients; although the surgeons drew on routine postoperative consultations, the set reflects clinician perception of patient concerns, and questions that patients ask online but not in clinic may be underrepresented, which limits external validity. Third, the standardized prompt explicitly requested warning signs and advice on seeking care, so the patient safety scores reflect prompted rather than spontaneous safety behavior and are likely to overestimate the safety content of responses to unprompted patient questions; for the same reason, and because no patient outcomes, adverse events, or real-world use were studied, the findings characterize the safety-related content of responses rather than the clinical safety of the models. Fourth, both the questions and responses were assessed exclusively in English; therefore, the models' performance in other languages falls outside the scope of this study. Fifth, because the analysis was limited to postoperative questions regarding lumbar fusion surgery, the results may differ for other conditions such as lumbar disc herniation, decompression, or alternative spinal procedures. Sixth, the evaluation was conducted exclusively by expert surgeons; patient interpretations of the same responses may differ, highlighting the importance of incorporating patient perspectives in future research. Relatedly, the reference standard for accuracy combined published evidence with institutional practice and expert judgment, because no single guideline covers postoperative lumbar fusion counseling; for activity-related questions on which practice varies, accuracy ratings therefore carry an element of subjectivity, that is, reflected in the modest interrater reliability. Seventh, premium (paid) versions were not included, and the findings therefore reflect freely accessible versions used by the general public; although this was a deliberate methodological choice to reflect real-world usage, it should be noted that premium versions and larger models (e.g., Gemini Pro rather than Gemini Flash) may demonstrate superior performance; performance may also differ through the API or after routine model updates. Eighth, interrater reliability at the single-rater level was poor for all subdomains and remained below 0.50 for clarity, relevance, and patient safety even after averaging three raters; although averaging mitigates this problem for model-level comparisons, the subdomain results are less reproducible than the composite score, and conclusions drawn from them should be correspondingly cautious. Ninth, a pronounced ceiling effect was present, with 77% of individual accuracy ratings at the maximum value; the 4-point scales used here, while common in this literature, lack the sensitivity to discriminate between advanced models whose responses are all broadly acceptable, and finer-grained instruments (for example, itemized checklists of expected content or error taxonomies) would be better suited to detecting subtle but clinically meaningful differences. Tenth, with 30 questions as the unit of analysis the study was powered to detect only moderate pairwise differences, the significant effects observed were small, and the category-level and *post hoc* safety-screening analyses were exploratory and were added at revision; the category grouping itself was defined *a priori*, but the safety-screening criterion was *post hoc*. In addition, the readability indices were computed with a tool that treats every line break, bullet point, and heading as a sentence boundary; because the three models differ systematically in how they format their answers—in their use of lists, headings, and short lines—part of the between-model difference in the Flesch and Gunning Fog scores may reflect these formatting conventions rather than linguistic complexity alone, and the readability comparisons should be interpreted with this in mind. Finally, the modified DISCERN-based framework, although grounded in the original instrument, remains unvalidated, and the full rubric is provided in Supplementary Table 2 so that it can be reused and tested. Despite these limitations, the present study provides a current, comparative perspective on the performance of three large language models in responding to postoperative lumbar fusion patient queries and helps clarify the role and limitations of artificial intelligence in clinical practice.

## Conclusion

This study demonstrates that responses from contemporary large language models to frequently asked questions by patients following lumbar fusion surgery were generally rated as accurate by blinded expert raters using a standardized, safety-oriented prompt. Differences among the models were statistically detectable but small. After correction across the six outcome families, robust between-model differences remained only for quality and completeness, on which Gemini scored lower than ChatGPT and Claude; the lower clarity of Gemini relative to Claude was observed in within-domain pairwise testing and should be regarded as exploratory, and no model was shown to be superior in relevance or safety-related content. More readable responses did not necessarily correspond to higher quality or clinically safer information, and this model-level pattern was not explained by response length.

In exploratory question- and category-level analyses, the models performed well on guideline-driven and standardized queries but appeared more limited in contexts requiring individualized clinical judgment and risk stratification, with wound-related questions emerging as a shared weak point; these observations are hypothesis-generating and warrant confirmation in larger, prespecified analyses. These findings indicate that artificial intelligence may serve as a supportive adjunct in postoperative patient education; however, it cannot substitute for physician evaluation, particularly in high-risk scenarios. Because safety-related content was explicitly requested and no patient outcomes were assessed, the present results should not be read as evidence that these models are safe for unsupervised postoperative use.

Future research should assess within-model response variability through repeated querying, compare prompted with unprompted safety behavior, use finer-grained instruments that avoid ceiling effects, focus on improving the clinical reliability of these systems and achieve an optimal balance between patient-friendly communication and medical accuracy.

## Data Availability

The raw data supporting the conclusions of this article will be made available by the authors, without undue reservation.

## References

[1] Mobbs RJ Phan K Malham G Seex K Rao PJ. Lumbar interbody fusion: techniques, indications and comparison of interbody fusion options including PLIF, TLIF, MI-TLIF, OLIF/ATP, LLIF and ALIF. J Spine Surg. (2015) 1(1):2–18. 10.3978/j.issn.2414-469X.2015.10.05 27683674 PMC5039869

[2] Reid PC Morr S Kaiser MG. State of the union: a review of lumbar fusion indications and techniques for degenerative spine disease: JNSPG 75th anniversary invited review article. J Neurosurg Spine. (2019) 31(1):1–14. 10.3171/2019.4.SPINE18915 31261133

[3] Yavin D Casha S Wiebe S Feasby TE Clark C Isaacs A. Lumbar fusion for degenerative disease: a systematic review and meta-analysis. Neurosurgery. (2017) 80(5):701–15. 10.1093/neuros/nyw162 28327997

[4] Burgess LC Arundel J Wainwright TW. The effect of preoperative education on psychological, clinical and economic outcomes in elective spinal surgery: a systematic review. Healthcare. (2019) 7:48. 10.3390/healthcare7010048 30901875 PMC6473918

[5] Eastwood D Manson N Bigney E Darling M Richardson E Paixao R. Improving postoperative patient reported benefits and satisfaction following spinal fusion with a single preoperative education session. Spine J. (2019) 19(5):840–5. 10.1016/j.spinee.2018.11.010 30471460

[6] Archer KR Coronado RA Haug CM Vanston SW Devin CJ Fonnesbeck CJ. A comparative effectiveness trial of postoperative management for lumbar spine surgery: changing behavior through physical therapy (CBPT) study protocol. BMC Musculoskelet Disord. (2014) 15(1):325. 10.1186/1471-2474-15-325 25273991 PMC4192328

[7] Strøm J Nielsen CV Jørgensen LB Andersen NT Laursen M. A web-based platform to accommodate symptoms of anxiety and depression by featuring social interaction and animated information in patients undergoing lumbar spine fusion: a randomized clinical trial. Spine J. (2019) 19(5):827–39. 10.1016/j.spinee.2018.11.011 30500464

[8] Jackson KL Rumley J Griffith M Agochukwu U DeVine J. Correlating psychological comorbidities and outcomes after spine surgery. Global Spine J. (2020) 10(7):929–39. 10.1177/2192568219886595 32905726 PMC7485071

[9] Yun HS Bickmore T. Online health information–seeking in the era of large language models: cross-sectional web-based survey study. J Med Internet Res. (2025) 27:e68560. 10.2196/68560 40163112 PMC11997521

[10] Akinleye SD Garofolo-Gonzalez G Montuori M Culbertson MD Hashem J Edelstein DM. Readability of the most commonly accessed online patient education materials pertaining to pathology of the hand. Hand. (2018) 13(6):705–14. 10.1177/1558944717726138 28836453 PMC6300184

[11] Vives M Young L Sabharwal S. Readability of spine-related patient education materials from subspecialty organization and spine practitioner websites. Spine. (2009) 34(25):2826–31. 10.1097/BRS.0b013e3181b4bb0c 19910867

[12] Powell J Inglis N Ronnie J Large S. The characteristics and motivations of online health information seekers: cross-sectional survey and qualitative interview study. J Med Internet Res. (2011) 13(1):e20. 10.2196/jmir.1600 21345783 PMC3221342

[13] Lang S Vitale J Fekete TF Haschtmann D Reitmeir R Ropelato M. Are large language models valid tools for patient information on lumbar disc herniation? The spine surgeons’ perspective. Brain Spine. (2024) 4:102804. 10.1016/j.bas.2024.102804 38706800 PMC11067000

[14] Sallam M. ChatGPT utility in healthcare education, research, and practice: systematic review on the promising perspectives and valid concerns. Healthcare. (2023) 11:887. 10.3390/healthcare11060887 36981544 PMC10048148

[15] Haupt CE Marks M. AI-generated medical advice—GPT and beyond. JAMA. (2023) 329(16):1349–50. 10.1001/jama.2023.5321 36972070

[16] Singhal K Azizi S Tu T Mahdavi SS Wei J Chung HW. Large language models encode clinical knowledge. Nature. (2023) 620(7972):172–80. 10.1038/s41586-023-06291-2 37438534 PMC10396962

[17] Yang R Tan TF Lu W Thirunavukarasu AJ Ting DSW Liu N. Large language models in health care: development, applications, and challenges. Health Care Sci. (2023) 2(4):255–63. 10.1002/hcs2.61 38939520 PMC11080827

[18] Ayers JW Poliak A Dredze M Leas EC Zhu Z Kelley JB. Comparing physician and artificial intelligence chatbot responses to patient questions posted to a public social media forum. JAMA Intern Med. (2023) 183(6):589–96. 10.1001/jamainternmed.2023.1838 37115527 PMC10148230

[19] Aydin S Karabacak M Vlachos V Margetis K. Large language models in patient education: a scoping review of applications in medicine. Front Med. (2024) 11:1477898. 10.3389/fmed.2024.1477898 PMC11554522 39534227

[20] Lang SP Yoseph ET Gonzalez-Suarez AD Kim R Fatemi P Wagner K. Analyzing large language models’ responses to common lumbar spine fusion surgery questions: a comparison between ChatGPT and bard. Neurospine. (2024) 21(2):633–41. 10.14245/ns.2448098.049 38955533 PMC11224745

[21] Mejia MR Arroyave JS Saturno M Ndjonko LCM Zaidat B Rajjoub R. Use of ChatGPT for determining clinical and surgical treatment of lumbar disc herniation with radiculopathy: a north American spine society guideline comparison. Neurospine. (2024) 21(1):149–58. 10.14245/ns.2347052.526 38291746 PMC10992643

[22] Hernandez F Guizar R Avetisian H Abdou MA Karakash WJ Ton A. Evaluating the accuracy and readability of ChatGPT in addressing patient queries on adult spinal deformity surgery. Global Spine J. (2026) 16(2):904–11. 10.1177/21925682251360655 40643892 PMC12254131

[23] Sarikonda A Abishek R Isch EL Momin AA Self M Sambangi A. Assessing the clinical appropriateness and practical utility of ChatGPT as an educational resource for patients considering minimally invasive spine surgery. Cureus. (2024) 16(10):e71105. 10.7759/cureus.71105 39525124 PMC11548952

[24] Wójcik D Adamiak O Czerepak G Tokarczuk O Szalewski L. Comparing the performance of ChatGPT, Gemini, and Claude in English and Polish on medical examinations. Sci Rep. (2025) 15(1):33083. 10.1038/s41598-025-17030-0 PMC12706083 41398197

[25] Karagöz B Bayrak HC Keçeci T. Comparison of ChatGPT-5 and DeepSeek V3 for artificial intelligence-assisted patient education in foot and ankle disorders. J Am Podiatr Med Assoc. (2026) 116:33. 10.3390/japma116030033 42200996

[26] Keçeci T Karagöz B. Can large language models follow guidelines? A comparative study of ChatGPT-4o and DeepSeek AI in clavicle fracture management based on AAOS recommendations. BMC Med Inform Decis Mak. (2025) 25:350. 10.1186/s12911-025-03202-5 PMC12482356 41023674

[27] Bayrak HC Karagöz B Bayrak Ö. Comparative evaluation of large language model-based chatbots in a septic arthritis scenario: ChatGPT, Claude, and Perplexity. Acta Orthop Traumatol Turc. (2025) 59:415–20. 10.5152/j.aott.2025.25428 41578826 PMC12839511

[28] Flesch R. A new readability yardstick. J Appl Psychol. (1948) 32(3):221–33. 10.1037/h0057532 18867058

[29] Subramanian T Shahi P Araghi K Mayaan O Amen TB Iyer S. Using artificial intelligence to answer common patient-focused questions in minimally invasive spine surgery. J Bone Joint Surg. (2023) 105(20):1649–53. 10.2106/JBJS.23.00043 37247341

[30] Walker HL Ghani S Kuemmerli C Nebiker CA Müller BP Raptis DA. Reliability of medical information provided by ChatGPT: assessment against clinical guidelines and patient information quality instrument. J Med Internet Res. (2023) 25:e47479. 10.2196/47479 37389908 PMC10365578

[31] Charnock D Shepperd S Needham G Gann R. DISCERN: an instrument for judging the quality of written consumer health information on treatment choices. J Epidemiol Community Health. (1999) 53(2):105–11. 10.1136/jech.53.2.105 10396471 PMC1756830

[32] Kincaid JP Fishburne RP Jr Rogers RL Chissom BS. Derivation of New Readability Formulas (Automated Readability Index, Fog Count and Flesch Reading Ease Formula) for Navy Enlisted Personnel. (1975).

[33] Gunning R. The Technique of Clear Writing. New York: McGraw-Hill (1952).

[34] Koo TK Li MY. A guideline of selecting and reporting intraclass correlation coefficients for reliability research. J Chiropr Med. (2016) 15(2):155–63. 10.1016/j.jcm.2016.02.012 27330520 PMC4913118

[35] Weiss BD. Health Literacy: A Manual for Clinicians. Chicago, IL: American Medical Association Foundation and American Medical Association (2003).

[36] Kirchner GJ Kim RY Weddle JB Bible JE. Can artificial intelligence improve the readability of patient education materials? Clin Orthop Relat Res. (2023) 481(11):2260–7. 10.1097/CORR.0000000000002668 37116006 PMC10566892

