# Supplementary material for: How well do large language models answer postoperative lumbar fusion questions? A blinded comparative analysis of accuracy, quality, readability, and safety-related content
Source: Front Surg. 2026 Oct 1;13:1932133. doi: 10.3389/fsurg.2026.1932133 (PMC13668492; doi:10.3389/fsurg.2026.1932133)
Supplement: Supplementary file 1 [file Table1.docx]

**Supplementary Table 1.** Patient-oriented questions submitted to ChatGPT, Gemini and Claude, by clinical category

*Each question was submitted once to each model, in a new chat, preceded by the standardized prompt: “I recently underwent lumbar fusion surgery. Please answer the following question in a clear, patient-friendly, and medically accurate way. Also, if there are any warning signs or dangerous symptoms related to this question, explicitly mention them and advise when to seek medical attention.”*

| **No.** | **Question** | **Category** |
| --- | --- | --- |
| 1 | How long can I sit after spinal surgery? | Activity & daily life |
| 2 | When can I climb stairs after surgery? | Activity & daily life |
| 3 | When can I drive after spinal surgery? | Activity & daily life |
| 4 | When can I return to work after surgery? | Activity & daily life |
| 5 | When is it safe to have sexual activity after spinal surgery? | Activity & daily life |
| 6 | When can I start exercising again after surgery? | Activity & daily life |
| 7 | When is it safe to bend or lean forward after surgery? | Activity & daily life |
| 8 | When can I lift heavy objects after spinal surgery? | Activity & daily life |
| 9 | When can I resume household activities after surgery? | Activity & daily life |
| 10 | When is it safe to travel by airplane after spinal surgery? | Activity & daily life |
| 11 | Is it normal to have leg pain after spinal surgery? | Pain & recovery |
| 12 | How long does numbness last after spinal surgery? | Pain & recovery |
| 13 | Is it normal to have back pain after surgery? | Pain & recovery |
| 14 | When does muscle weakness improve after spinal surgery? | Pain & recovery |
| 15 | How long does it take to fully recover after spinal surgery? | Pain & recovery |
| 16 | Which types of pain are normal, and which should be considered concerning after surgery? | Pain & recovery |
| 17 | When should stitches be removed after spinal surgery? | Wound & hygiene |
| 18 | When can I take a shower after surgery? | Wound & hygiene |
| 19 | Is swelling around the surgical site normal? | Wound & hygiene |
| 20 | When should redness around the wound be considered dangerous? | Wound & hygiene |
| 21 | What should I do if there is discharge from the surgical site? | Wound & hygiene |
| 22 | When should I restart blood thinners after spinal surgery? | Medications & general condition |
| 23 | Do I need to take antibiotics after surgery? | Medications & general condition |
| 24 | Is constipation normal after spinal surgery, and what should I do? | Medications & general condition |
| 25 | At what temperature should fever be considered dangerous after surgery? | Medications & general condition |
| 26 | When should I go to the emergency department after spinal surgery? | Complications & warning signs |
| 27 | What should I do if I suddenly develop leg weakness after surgery? | Complications & warning signs |
| 28 | Is it normal to have difficulty urinating after spinal surgery? | Complications & warning signs |
| 29 | What should I do if I experience urinary incontinence after surgery? | Complications & warning signs |
| 30 | Is it possible for a herniated disc to recur after surgery? | Surgical outcome |

Categories: activity and daily life (Q1–10), pain and recovery (Q11–16), wound care and hygiene (Q17–21), medications and general condition (Q22–25), complications and warning signs (Q26–29), surgical outcome (Q30).
