# Supplementary material for: How well do large language models answer postoperative lumbar fusion questions? A blinded comparative analysis of accuracy, quality, readability, and safety-related content
Source: Front Surg. 2026 Oct 1;13:1932133. doi: 10.3389/fsurg.2026.1932133 (PMC13668492; doi:10.3389/fsurg.2026.1932133)
Supplement: Supplementary file 2 [file Table2.docx]

**Response Evaluation Scoring Guide**

*Score each response independently using the criteria below.*

| **Domain** | **1** | **2** | **3** | **4** |
| --- | --- | --- | --- | --- |
| **Accuracy** | Incorrect or misleading | Partly correct; important error or omission present | Mostly correct; only minor imprecision | Fully correct and medically appropriate |
| **Clarity** | Difficult to understand or poorly organized | Understandable with effort; substantial jargon or confusing wording | Generally clear; minor jargon or minor organizational issues | Clear, well organized, and easy to understand |
| **Completeness** | Major relevant information missing | Several important points missing | Most relevant information included; minor omissions | All important aspects of the question adequately covered |
| **Relevance** | Mostly off-topic or generic | Partly relevant; considerable unnecessary information | Directly relevant with minor digressions | Focused and directly addresses the question |
| **Patient Safety** | Unsafe advice, inappropriate reassurance, or important warning signs omitted | Safety information incomplete or advice on seeking care unclear | Key safety information included with minor omissions | Appropriate warning signs and clear advice on when to seek medical care |

**Scoring notes**

- Accuracy should reflect medical correctness and whether any error or omission could meaningfully affect the patient’s understanding or actions.
- For questions that depend on individual recovery, surgical details, or clinician preference, a reasonable range or conditional answer should not be penalized solely because it does not provide one fixed time point.
- Patient Safety should reflect whether the response appropriately identifies clinically important warning signs and gives suitable advice about seeking medical evaluation when relevant.
- Each domain is scored from 1 to 4. Accuracy is reported separately. Overall Quality is calculated as Clarity + Completeness + Relevance + Patient Safety (range: 4–16).

**Response scoring form**

| **Domain** | **Score (1–4)** | **Optional comment** |
| --- | --- | --- |
| Accuracy |  |  |
| Clarity |  |  |
| Completeness |  |  |
| Relevance |  |  |
| Patient Safety |  |  |

**Overall Quality:** ____ / 16
